# Supplementary material for: Crystal structure of dopamine D1 receptor in complex with G protein and a non-catechol agonist
Source: Nat Commun. 2021 Jun 3;12:3305. doi: 10.1038/s41467-021-23519-9 (PMC8175458; doi:10.1038/s41467-021-23519-9)
Supplement: Supplementary file 1 — Supplementary Information [file 41467_2021_23519_MOESM1_ESM.pdf]

Supplementary Materials

Supplementary Figure 1

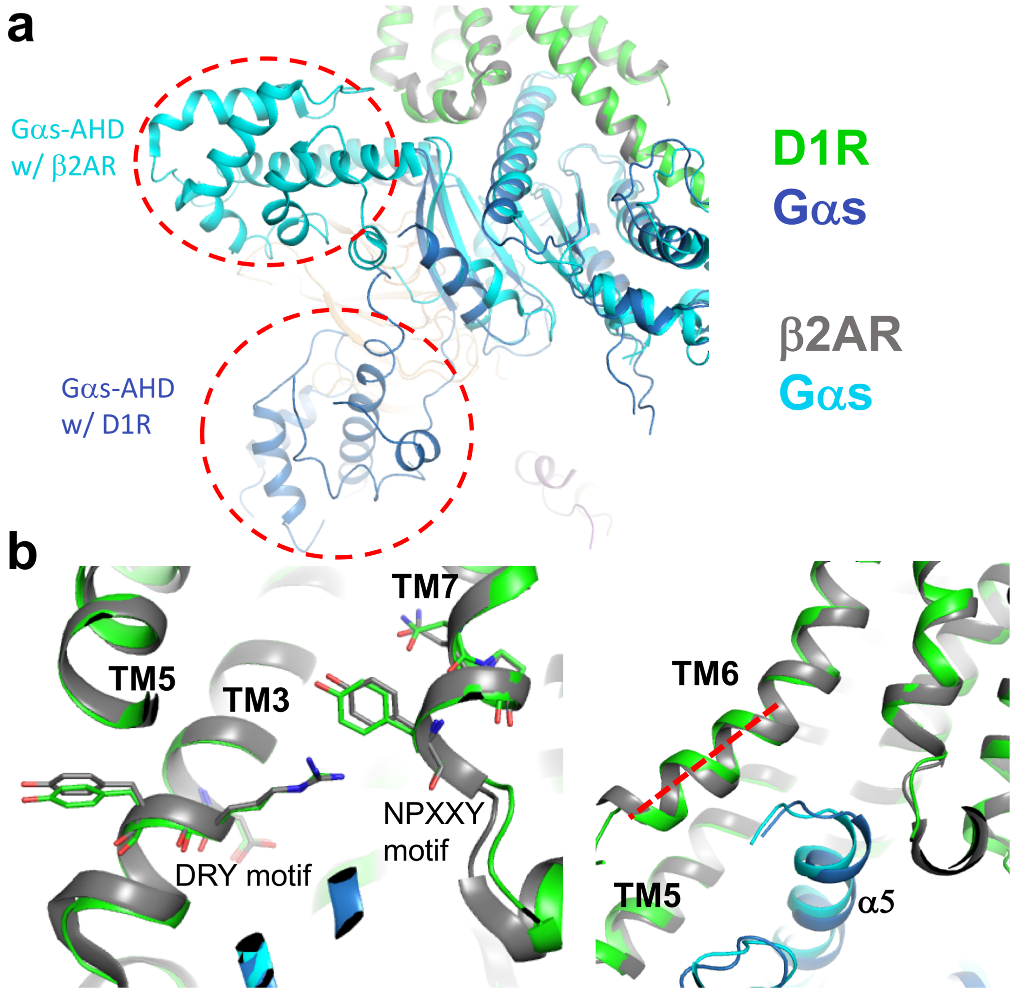

Supplementary Figure 1. Overall structure feature of D1R-Gs. **a** The Gαs-AHD in D1R-Gs (D1R in green, dark blue for Gαs) and β2AR-Gs (β2AR in gray, Gαs in cyan, PDB ID: 3SN6) structures are in different positions. **b** The conformation of the DRY and NPXXY motifs (left panel) and TM6 (right panel) in D1R-Gs and β2AR-Gs structures.

Supplementary Figure 2

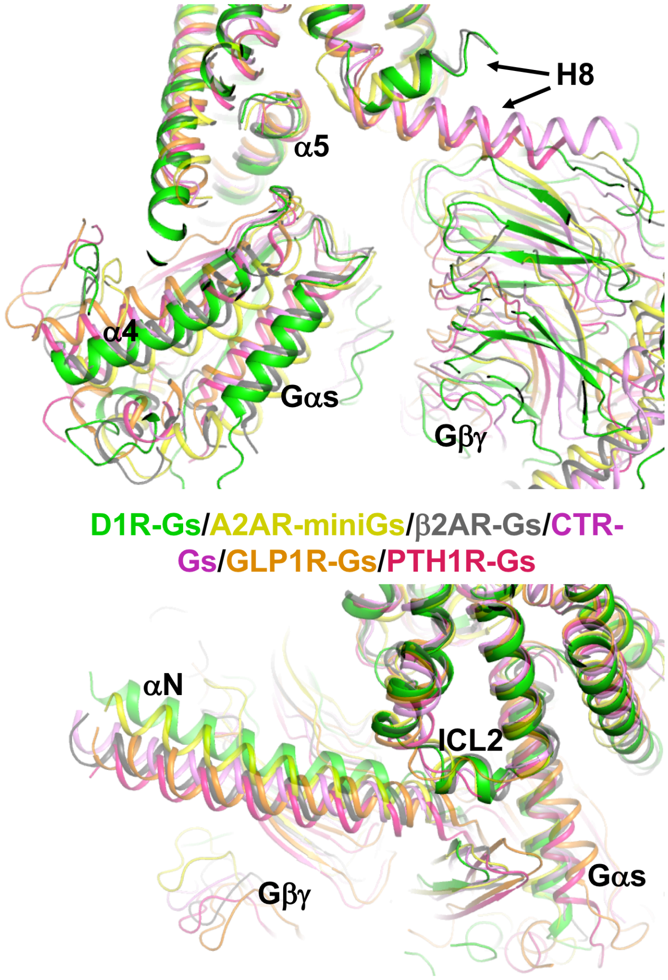

Supplementary Figure 2. The relative orientation of GPCRs coupled to Gs. D1R-Gs: green; β2AR-Gs (gray, PDB ID: 3SN6); A2AR-miniGs (yellow, PDB ID: 6GDG); CTR-Gs (magenta, PDB ID: 5UZ7); GLP1R-Gs (orange, PDB ID: 5VAI); PTH1R-Gs (hotpink, PDB ID: 6NBF). Upper panel: different orientation of H8 in receptors; lower panel: different conformation of ICL2 in receptors.

Supplementary Figure 3

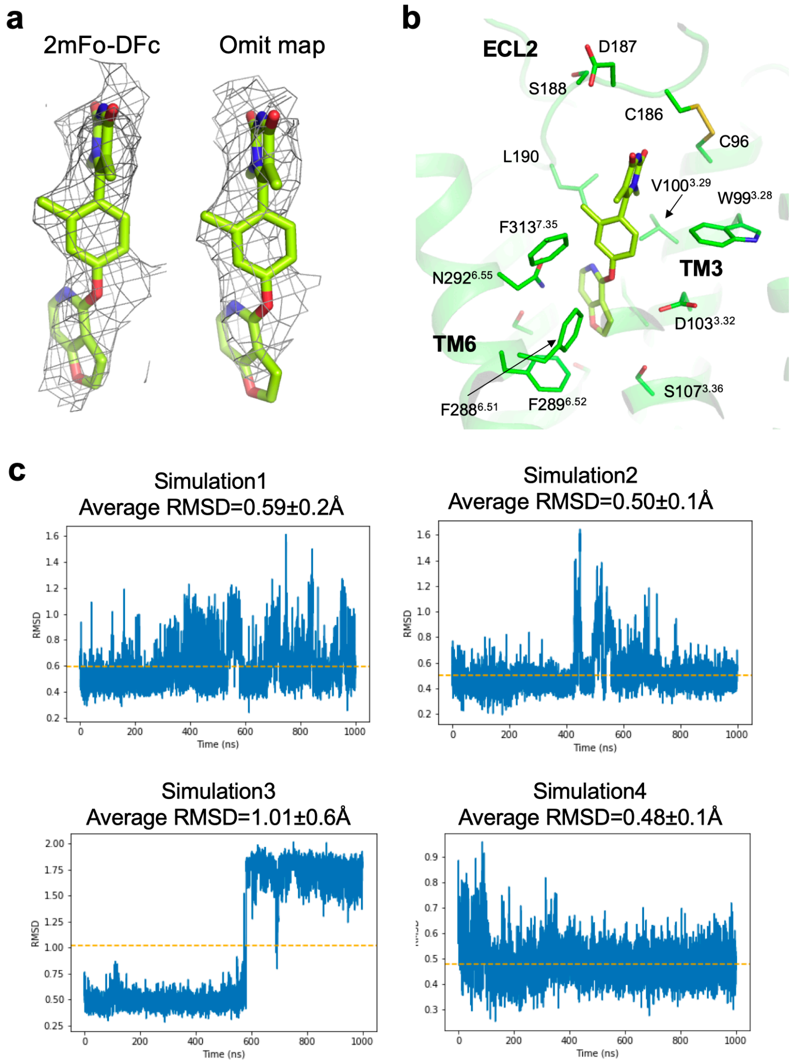

Supplementary Figure 3. The Compound 1 binding pocket. **a** 2mFo-Dfc map (after refinement, left) and composite omit map (right) for the ligand, contoured at 1.0σ. **b** MD simulation of Compound 1 binding to D1R. Hydrogen bonds are shown in black dashed lines. This is a representation of four independent 1μs MD simulations. **c** RMSD plots monitoring the stability of the agonist in the orthosteric site in four independent 1μs MD simulations. Crystal structure pose is used as zero time point. The resulting average RMSD is 0.64 ± 0.2 Å (S.D.) confirming the stable binding pose of the Compound 1 in the orthosteric site.

Supplementary Figure 4

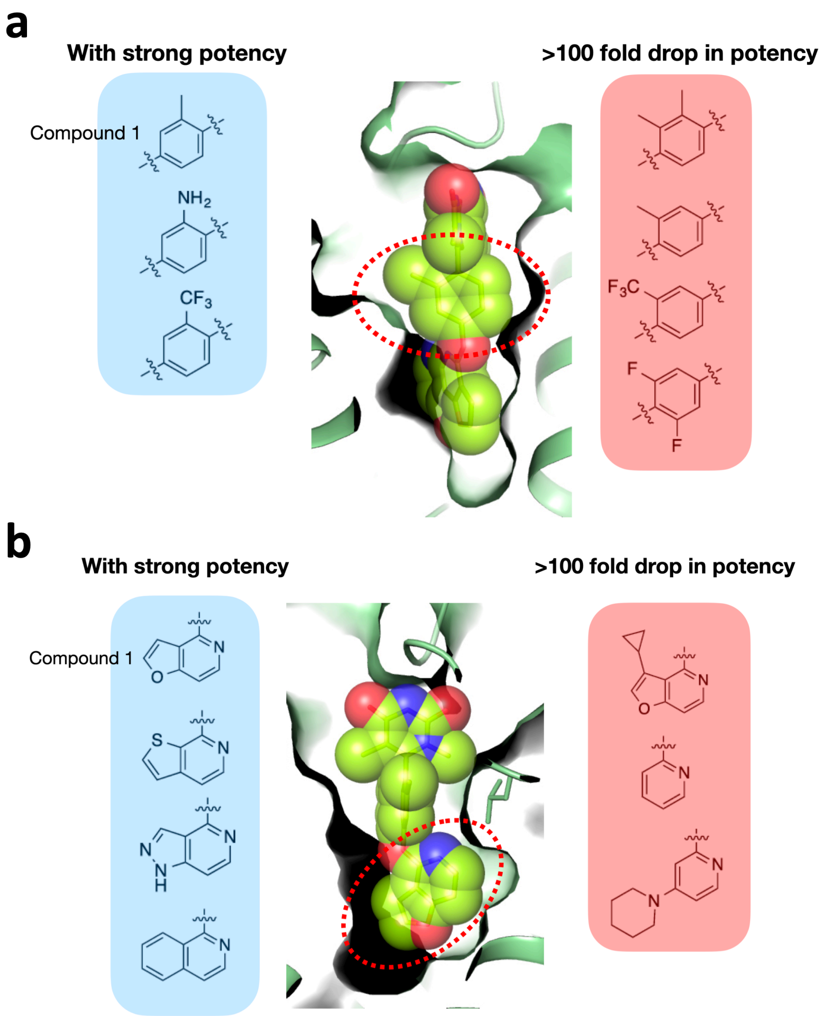

Supplementary Figure 4. Structure-activity relationship of Compound 1 scaffold explained by the D1R-Gs structure. Analogs with various substitute groups at the central phenoxy ring or the furo-pyridine ring (indicated by dotted red oval) of Compound 1 are shown in **a** and **b**, respectively. The potency of the analogs was reported in citation 6.

Supplementary Figure 5

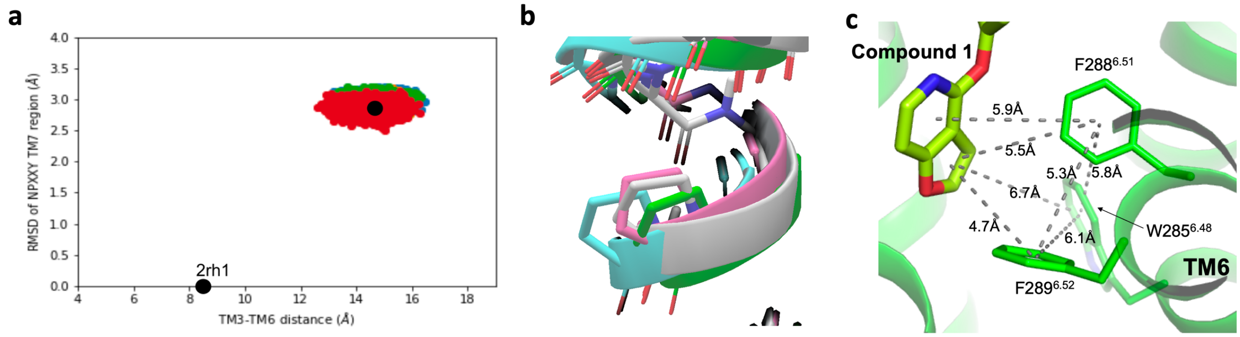

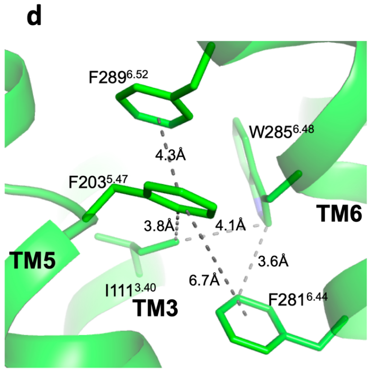

e

Residue/ligand pair	Average Distance(Å)
Phe288$^{6.51}$ to Compound 1	3.7±0.6
Phe289$^{6.52}$ to Compound 1	3±0.3
Trp285$^{6.48}$ to Compound 1	5.9±0.8
Trp285$^{6.48}$ to Phe281$^{6.44}$	2.1±0.2
Ile111$^{3.40}$ to Phe281$^{6.44}$	2.4±0.5
Phe203$^{5.47}$ to Phe281$^{6.44}$	4.4±0.6
Phe203$^{5.47}$ to Phe289$^{6.52}$	2.7±0.2
Phe203$^{5.47}$ to Ile111$^{3.40}$	2.4±0.3

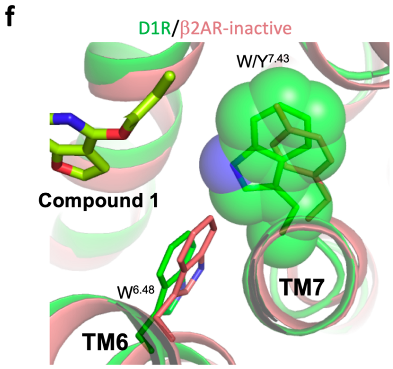

Supplementary Figure 5. Structural insights into D1R activation by Compound 1. **a** 2D plot showing the conformational space explored by the receptor bound to the agonist Compound 1 in 4 independent 1 μs MD simulations as a function of the TM3-TM6 distance (considering the Cα atoms of Arg121$^{3.50}$ and Val270$^{6.33}$) and the RMSD from inactive-state of the NPXXY motif of TM7 (taking the β2AR inactive-state structure as reference point, labeled in figure, PDB ID: 2RH1). Each dot corresponds to an MD frame (structure) and the dots are colored depending on the simulation they belong to. The black dot in the middle of colorful dot cloud indicates the conformation of the initial MD structure. **b** Position of Pro$^{5.50}$ of D1R-Gs crystal structure (white), D1R after MD simulation (green), β2AR-Gs (cyan, PDB ID: 3SN6), and β2AR inactive-state (pink, PDB ID: 2RH1) structures. The representative conformation of D1R after MD simulation is shown. The side chains of residues are shown in stick. **c** Distances between Compound 1 and critical residues on D1R that likely initiate the conformation changes leading to receptor activation. Aromatic rings with their centroids' distance smaller than 7Å and proper dihedral angles are considered to be able to form aromatic interactions between them (1). All distances are labeled in unit Å. **d** The distances between residues from the orthosteric pocket to the PIF motif. **e** The distances between Compound 1 and critical residues, or residues involved in D1R activation during MD simulations. The distance values are the average of 5 independent 50 ns MD simulations (average ± S.D., Å) between the closest atoms of a respective residue and Compound 1. All distances are stable throughout the simulation and the results indicate stable interactions between each pair. **f** Rotamer of Trp7.43 (shown in stick and spheres) in D1R (green) is not compatible with Trp6.48 side chain position estimated by aligning the β2AR inactive-state structure (pink, PDB ID: 2RH1) with the D1R structure.

Supplementary Figure 6

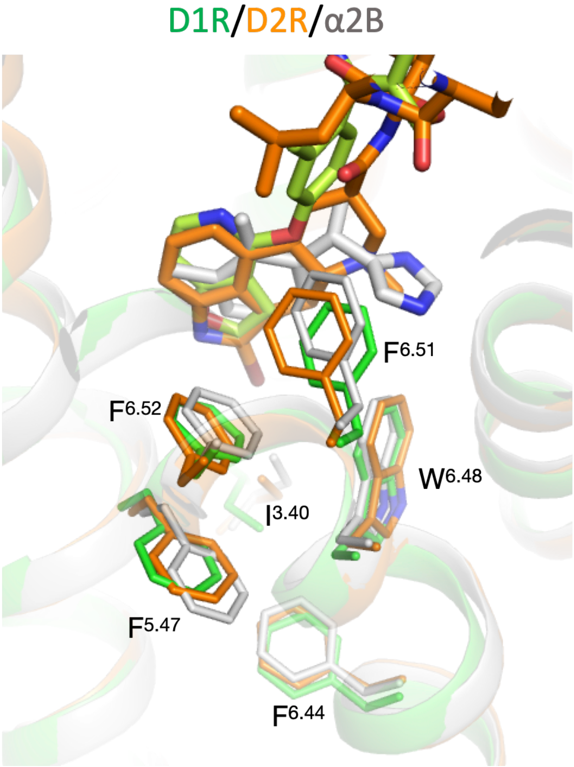

Supplementary Figure 6. The network of aromatic residues that are critical for the activation of D1R and other aminergic receptors. The structure of D1R (green), D2R (orange, PDB ID: 6VMS) and α2B receptor (silver gray, PDB ID: 6K41) bound to non-catechol agonists have been aligned. The bound agonists, and the network of aromatic residues are shown in sticks.

Supplementary Figure 7

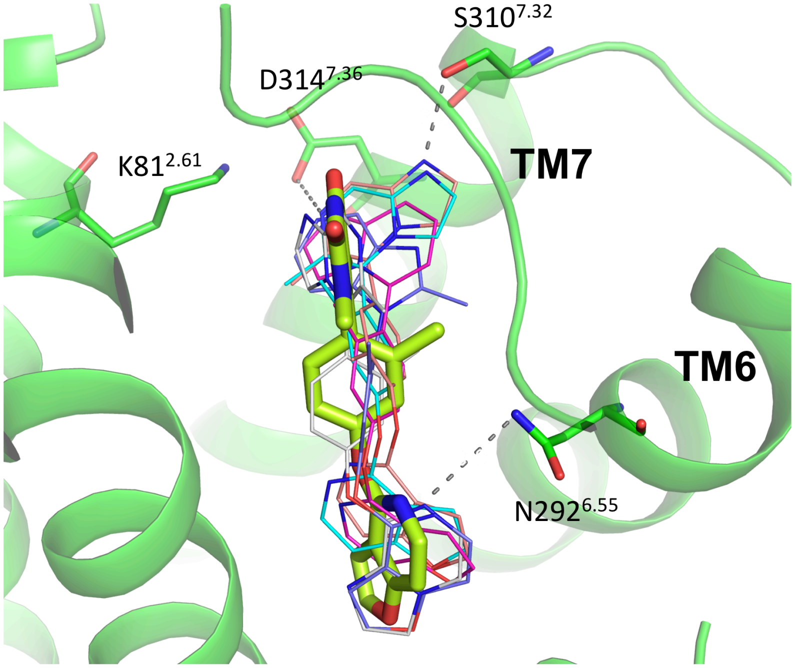

Supplementary Figure 7. Docking poses of reported Compound 1 analogs to D1R structure. D1R from the crystal structure is shown in green, while Compound 1 is shown in lime. Compound 1, Lys81$^{2.61}$, Asp314$^{7.36}$, Ser310$^{7.32}$, Asn292$^{6.55}$ are shown in stick, while Compound 1 analogs are shown in line. Analogs that are shown in cyan, magenta, salmon and gray are reported in (17) (named Compound 1, 4, 14, 31 in reference (17)). PF6142 (6) is shown in blue. Possible hydrogen bonds with distance smaller than 4Å are marked by dashed lines.

Supplementary Figure 8

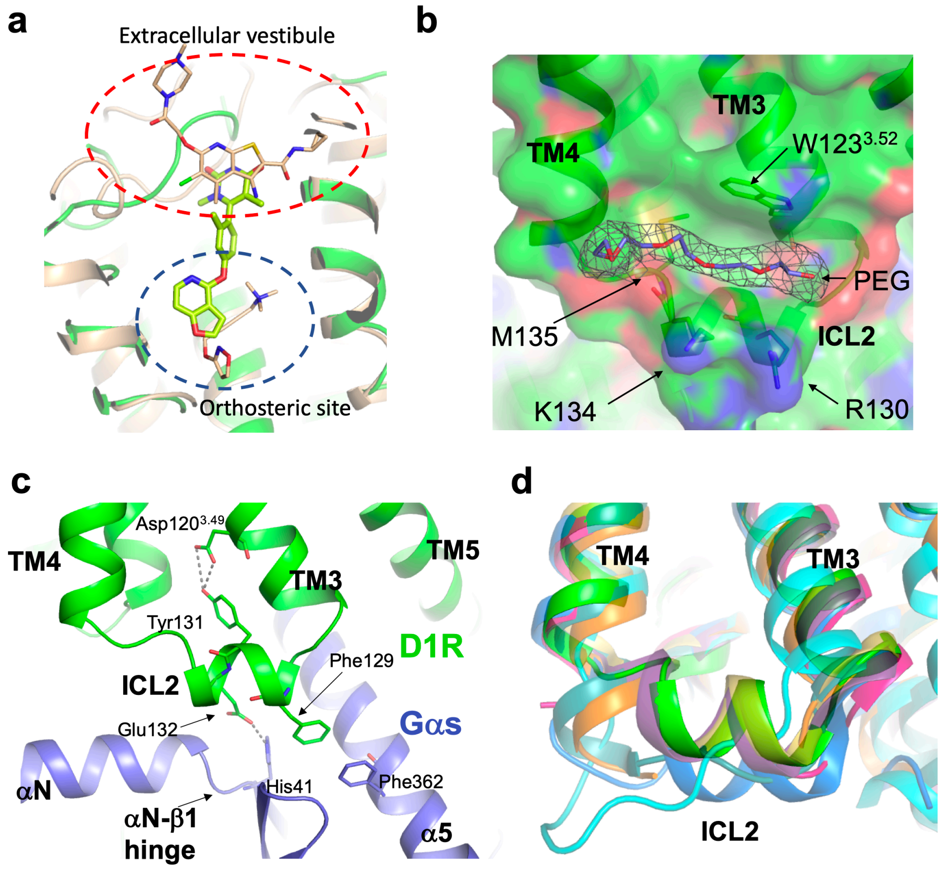

Supplementary Figure 8. a Compound 1 extends into the extracellular vestibule of D1R where allosteric modulators of other GPCRs have been observed. The D1R structure (green) is aligned with structure of the muscarinic M2 receptor in complex with agonist Iperoxo and PAM LY2119620 (beige, PDB ID: 4MQT). The orthosteric site and allosteric site defined by the position of Iperoxo and LY2119620 are delineated with dashed blue and red lines, respectively. **b** Surface view of the TM3-ICL2-TM4 region. A continuous density in the 2mFo-DFc map is shown in mesh, contoured at 1.0σ. A PEG400 molecule (blue) in crystallization environment is modeled to fit the density. **c** The helical conformation of ICL2 is critical for stabilizing the DRY motif in the active conformation and is seen to interact with Gs. Residues involved in these interactions are shown in stick, and hydrogen bonds are depicted with dash lines. D1R is shown in green and Gαs is shown in blue. **d** Conformation of ICL2 in aminergic receptors. D1R: green; D2R: orange (PDB ID: 6CM4); 5-HT2C, magenta (PDB ID: 6BQG); 5-HT1B, yellow (PDB ID: 4IAR); β1AR, purple (PDB ID: 2Y03); D4R, cyan (PDB ID: 5WIV); D3R, blue (PDB ID: 3PBL, chain A); 5-HT2B, dark green (PDB ID: 4IB4). ICL2 in the inactive or intermediate conformation of these receptors adopts a loop conformation or becomes disordered, except when directly involved in crystal packing, as is the case with β1AR, 5-HT1B and one monomer in the asymmetric unit of the D3R crystal structure.

Supplementary Figure 9

a

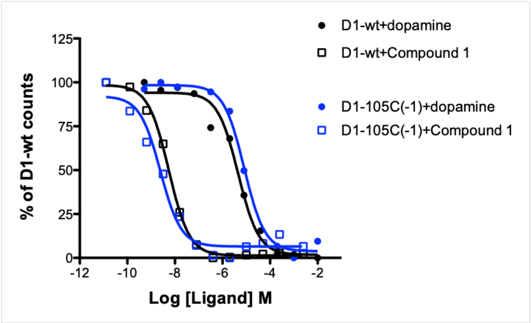

Construct	Ligand	Ki
D1-wt	dopamine	2.3μM
D1-wt	Compound 1	2.8nM
D1-105C(-1)	dopamine	4.1μM
D1-105C(-1)	Compound 1	1.2nM

b

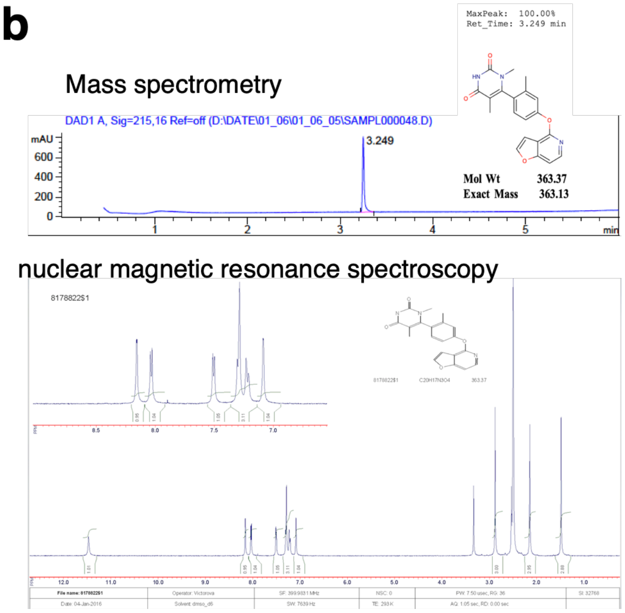

Supplementary Figure 9. Validation of the crystallization construct and ligand. **a** Validation of crystallization construct. Competition binding assay was carried out with 1nM of ^{3}H-SCH23390 and insect cell membranes expressing respective version of D1R. Increasing concentrations of dopamine or Compound 1 were added to test their affinity with wild-type D1R (D1-wt) or the crystallization construct (D1-105C(-1)). The results are listed in the table. The result shown is a representative of two independent experiments. **b** Validation of synthesized Compound 1 by mass spectrometry and nuclear magnetic resonance spectroscopy. The results from both spectrum indicated that the product is in full accordance with the chemical structure showing no side products.

Supplementary Table 1. Data collection and structural refinement statistics

**D1R-Gs-Compound 1 (PDB ID: 7JOZ)**	
Source, wavelength	APS 23ID-D, 1.0Å
Number of crystals	15
Space group	$P2_122_1$
Cell parameters	
a, *b*, *c* (Å)	99.2, 144.3, 147.1
α, β, γ (°)	90.0, 90.0, 90.0
Resolution (Å)	49.0–3.8 (4.0–3.8)
Average redundancy	3.6 (3.7)
Average $I/\sigma(I)$	4.7 (1.3)
Completeness (%)	94.3 (93.1)
R_{merge} (%)	18.9 (115.9)
R_{pim} (%)	11.6 (65.2)
$CC_{1/2}$	0.99 (0.40)
**Refinement**	
Unique reflections used for refinement	19954
R_{work}/R_{free} (%)	25.4/29.7
Free R percentage (%)	5.3
Average B factors (Å^2)	
Overall	140.0
D1R	154.0
Compound 1	138.4
R.m.s. deviation	
Bond length (Å)	0.002
Bond angles (°)	0.525
Ramachandran favored (%)	91.9
Ramachandran outliers (%)	0.0

* Highest shell statistics are in parentheses.

Reference

1. R. Anjana *et al.*, Aromatic-aromatic interactions in structures of proteins and protein-DNA complexes: a study based on orientation and distance. *Bioinformation* **8**, 1220-1224 (2012).
