# Supplementary material for: Crystal structure of dopamine D1 receptor in complex with G protein and a non-catechol agonist
Source: Nat Commun. 2021 Jun 3;12:3305. doi: 10.1038/s41467-021-23519-9 (PMC8175458; doi:10.1038/s41467-021-23519-9)
Supplement: Supplementary file 3 — Reporting Summary [file 41467_2021_23519_MOESM3_ESM.pdf]

nature research

Corresponding author(s): Brian Kobilka

Last updated by author(s): Apr 8, 2021

Reporting Summary

Nature Research wishes to improve the reproducibility of the work that we publish. This form provides structure for consistency and transparency in reporting. For further information on Nature Research policies, see our Editorial Policies and the Editorial Policy Checklist.

Statistics

For all statistical analyses, confirm that the following items are present in the figure legend, table legend, main text, or Methods section.

n/a	Confirmed	
☐	☒	The exact sample size (n) for each experimental group/condition, given as a discrete number and unit of measurement
☐	☒	A statement on whether measurements were taken from distinct samples or whether the same sample was measured repeatedly
☒	☐	The statistical test(s) used AND whether they are one- or two-sided<br>*Only common tests should be described solely by name; describe more complex techniques in the Methods section.*
☒	☐	A description of all covariates tested
☒	☐	A description of any assumptions or corrections, such as tests of normality and adjustment for multiple comparisons
☐	☒	A full description of the statistical parameters including central tendency (e.g. means) or other basic estimates (e.g. regression coefficient) AND variation (e.g. standard deviation) or associated estimates of uncertainty (e.g. confidence intervals)
☒	☐	For null hypothesis testing, the test statistic (e.g. F, t, r) with confidence intervals, effect sizes, degrees of freedom and P value noted<br>*Give P values as exact values whenever suitable.*
☒	☐	For Bayesian analysis, information on the choice of priors and Markov chain Monte Carlo settings
☒	☐	For hierarchical and complex designs, identification of the appropriate level for tests and full reporting of outcomes
☒	☐	Estimates of effect sizes (e.g. Cohen's d, Pearson's r), indicating how they were calculated

Our web collection on statistics for biologists contains articles on many of the points above.

Software and code

Policy information about availability of computer code

Data collection	JBluIce (ID-D or ID-B version, v2015.1)
Data analysis	CCP4 package 6.1.0, including Phaser; Phenix Package 1.13; Prism5.0b

For manuscripts utilizing custom algorithms or software that are central to the research but not yet described in published literature, software must be made available to editors and reviewers. We strongly encourage code deposition in a community repository (e.g. GitHub). See the Nature Research guidelines for submitting code & software for further information.

Data

Policy information about availability of data

All manuscripts must include a data availability statement. This statement should provide the following information, where applicable:

- Accession codes, unique identifiers, or web links for publicly available datasets
- A list of figures that have associated raw data
- A description of any restrictions on data availability

Data supporting the findings of this manuscript are available from the corresponding authors upon reasonable request. Structure and data set in this work have been deposited in the Protein Data Bank under accession code PDB 7JOZ.
Compound 1 is not immediately available upon request. It will have to be resynthesized.

Field-specific reporting

Please select the one below that is the best fit for your research. If you are not sure, read the appropriate sections before making your selection.

- [x] Life sciences
- [ ] Behavioural & social sciences
- [ ] Ecological, evolutionary & environmental sciences

For a reference copy of the document with all sections, see nature.com/documents/nr-reporting-summary-flat.pdf

Life sciences study design

All studies must disclose on these points even when the disclosure is negative.

Sample size	The curves shown are typical from one experiment with triplicate determinations, repeated at least twice.
Data exclusions	No data were excluded.
Replication	All experimental findings were reliably reproduced.
Randomization	Randomization was not required since no animal or human subjects are involved.
Blinding	Blinding was not required, since no animal or human subjects are involved and no human judgment was involved in generating the data.

Reporting for specific materials, systems and methods

We require information from authors about some types of materials, experimental systems and methods used in many studies. Here, indicate whether each material, system or method listed is relevant to your study. If you are not sure if a list item applies to your research, read the appropriate section before selecting a response.

Materials & experimental systems

n/a	Involved in the study	
[ ]	[x]	Antibodies
[ ]	[x]	Eukaryotic cell lines
[x]	[ ]	Palaeontology and archaeology
[x]	[ ]	Animals and other organisms
[x]	[ ]	Human research participants
[x]	[ ]	Clinical data
[x]	[ ]	Dual use research of concern

Methods

n/a	Involved in the study	
[x]	[ ]	ChIP-seq
[x]	[ ]	Flow cytometry
[x]	[ ]	MRI-based neuroimaging

Antibodies

Antibodies used	llama nanobody 35 was used to stabilize the D1-Gs complex, not for probing purposes. It was originally described in Rasmussen et al. Nature, 477, 549-555 (2011). The expression plasmid for nanobody 35 was obtained from the Kobilka lab at Stanford University.
Validation	The structure of the D1-Gs complex reveals that nanobody 35 bound to the interface of the alpha and beta subunits of Gs as expected. Nanobody 35 was not used for probing purposes, therefore, no further validation was needed.

Eukaryotic cell lines

Policy information about cell lines

Cell line source(s)	LMtk (Ltk-) mouse fibroblast cells expressing the dopamine D1 receptor (BioSignal Inc, Montreal, Canada, now Perkin Elmer)
Authentication	Cells were authenticated by fingerprinting and p-e presse proteins by sequencing .
Mycoplasma contamination	Regular mycoplasma tests were performed by UCB and by suppliers. The cells used for the data were tested negative.
Commonly misidentified lines (See ICLAC register)	No commonly misidentified cell lines were used in the study.
